# High-Resolution Microstructure Analysis of Cork Spot Disordered Pear Fruit “Akizuki” (*Pyrus pyrifolia* Nakai) Using X-Ray CT

**DOI:** 10.3389/fpls.2021.715124

**Published:** 2021-08-16

**Authors:** Zhenhua Cui, Nannan Wang, Yanxin Duan, Xinrui Xu, Ran Wang, Shaoling Zhang, Chunhui Ma

**Affiliations:** ^1^Department of Horticulture, Qingdao Agricultural University, Qingdao, China; ^2^Sanying Precision Instruments Co., Ltd., Tianjin, China; ^3^Department of Horticulture, State Key Laboratory of Crop Genetics and Germplasm Enhancement, Nanjing Agricultural University, Nanjing, China

**Keywords:** “Akizuki”, Ca^2+^ transport, microstructure, X-ray CT scanning, cork spot disorder

## Abstract

Cork spot is one of the most damaging physiological disorders in pear fruit, causing considerable economic loss every year. However, the mechanism of cork spot occurrence requires further examination. In this study, X-ray CT scanning was applied to analyze the microstructure of pear fruit “Akizuki” (*Pyrus pyrifolia*), a cultivar susceptible to cork spot disorder, to elucidate the fruit texture alteration between healthy and cork spotted fruit. Results showed that cork spotted fruit had much higher porosity (9.37%) than healthy fruit (3.52%). Reconstructed three-dimensional (3D) network skeleton models showed highly branched pore channels in cork spotted fruit and a low degree of pore connectivity in healthy fruit. Even in areas of disordered fruit without cork spot, the pore throat diameter, pore length, and coordinated core number (i.e., 77, 160, and 16, respectively) were much higher than that of healthy fruit. The structure analysis of fruit core showed that core deformation only occurred in cork spotted fruit. A much more highly branched network was observed in cork spotted fruit cores compared with healthy fruit cores. High-resolution observation of flesh tissue directly demonstrated that pore size in cork spotted fruit (87 μm) was four times larger than that of healthy fruit (22 μm). Altered expression of genes related to Ca^2+^ transport and the uneven distribution of intracellular Ca^2+^ were also shown to associate with the development of cork spot disorder. Our results suggest that flesh tissue damage likely occurred prior to the initiation of cork spot. The dysfunction of long-distance and transmembrane Ca^2+^ transport channels could be responsible for the imbalanced distribution of Ca^2+^ inside the fruit, thus resulting in the development of cork spot.

## Introduction

“Akizuki” (*Pyrus pyrifolia*), a late-maturing pear cultivar with russet skin, was bred in Japan in 1988. “Akizuki” has a large fruit size, attractive fruit shape, and delicate flesh with high sugar content, and it is an important economic crop in China. However, “Akizuki” is susceptible to cork spot disorder, one of the major physiological disorders in pear. This disorder has threatened the Chinese pear industry since 2000 (Hayama et al., 2017; Duan et al., 2019). Cork spot is a preharvest disorder, initiated at 60–80 days after full bloom (DAFB), and develops until fruit harvest, depending on cultivar specificity (Cui et al., 2020). Fruit with cork spots has brownish flesh or grayish corky lesions beneath the fruit skin, and the flesh develops a bitter taste. In some cultivars, disordered fruit has a bumpy fruit surface, such as in “Chili” (*Pyrus bretschneideri*) (Cui et al., 2020). Higher lignin content and necrotic tissues are typically observed in cork spotted areas of flesh, which is believed to be caused by mineral nutrient imbalances (Facteau et al., 2010), such as in Ca, Mg, B, and K. Ca deficiency in the fruit was originally considered as the major reason for the development of cork spot (Mason and Welsh, 1970; Woodbridge, 1971), and Ca^2+^ spray on fruit before the harvest was believed to reduce the incidence of cork spot (Raese and Drake, 1993, 2006; Raese et al., 1995; Sharma et al., 2013). However, in our recent studies, we found higher Ca content in cork spotted tissues compared with healthy tissues, and free Ca^2+^ was enriched in the cell wall, while less Ca^2+^ was distributed in the cytoplasm in disordered fruit (Duan et al., 2019; Cui et al., 2020). Therefore, we proposed that the dysfunction of Ca^2+^ transmembrane transport and intracellular distribution in the fruit could initiate the development of cork spot disorder in pear fruit (Cui et al., 2020).

Fruit microstructure determines the mechanical and transport properties of tissues (Mebatsion et al., 2009). In pear, the intercellular spaces occupy as much as 5.1% of the total fruit volume, which is the most important pathway for gas diffusion (e.g., O_2_, CO_2_, and H_2_O) in plants (Kader, 2002). Meanwhile, the vascular system is responsible for water and nutrient transport from the plant to the fruit. Therefore, a complete void network and the vascular system are critical for the development of healthy fruit. In our previous research, we found brownish vascular tissues distributed in cork spotted “Chili” fruit, and spotted areas were close to necrotic vascular tissues (Cui et al., 2020), indicating that the change of fruit tissue structure is associated with the occurrence of this physiological disorder. However, traditional histological studies with two-dimensional sections are destructive to the fruit and can only observe on a single plane, which is unable to correctly render critical connectivity information such as small voids between cells, vascular capillaries, or cell walls.

X-ray CT, a three-dimensional (3D) visualization technique, has long been applied to non-destructively acquire the 3D images of the internal structure of agricultural commodities (Schoeman et al., 2016). X-ray CT recognizes internal structures based on differences in X-ray attenuation resulting from 3D variation in the composition of the material. This makes X-ray CT an excellent technique to visualize the porous structure of products on both qualitative and quantitative scales (Herremans et al., 2014a,b; Magwaza and Opara, 2014; Diels et al., 2017). In apple fruit, Janssen et al. (2020) found considerable variations of fruit porosity in different tissues based on the 3D connectivity of pores. Microstructural changes were observed in apple tissues with water core disorder with the help of X-ray scanning (Herremans et al., 2014a). X-ray CT was also used to classify fruit tissue as healthy or browning disordered at a successful rate of 97% (Herremans et al., 2013), as well as being used for the quantitative analysis of the 3D topology of the pore space in apple fruit (Mendoza et al., 2007). In pear, core breakdown disorder was analyzed using X-ray CT over time in storage (Lammertyn et al., 2003). Herremans et al. (2015) characterized 3D tissue anatomy at the level of single cells and intercellular spaces in pear using the X-ray CT analysis, demonstrating significant differences between genotypes in void and cell networks that relate to differences in aeration properties of tissues. X-ray CT was also applied to analyze and detect the mealiness in “Forelle” (*Pyrus communis*) (Muziri et al., 2016). In addition to the abovementioned studies, X-ray CT was also used to visualize the subsurface microstructure and void network connectivity in other various products, such as mango (Cantre et al., 2014b), pomegranate (Magwaza and Opara, 2014), kiwifruit (Cantre et al., 2014a), and cucumber (Kuroki et al., 2004). However, the application of X-ray CT to analyze the microstructure differences between healthy pear fruit and cork spot disordered pear fruit is very limited. Knowledge of pear fruit microstructure changes in a quantitative and qualitative 3D view is important for a better understanding of the occurrence of cork spot disorder. Recent research (Duan et al., 2020) first attempted to non-destructively distinguish between healthy and cork spot disordered pear “Chili” (*P. bretschneideri*) and to preliminarily analyze the porous differences between them using X-ray CT. However, more information and investigation on the microstructure changes in cork spotted pear fruit are still needed to explore the mechanism of the development of cork spot disorder. Therefore, the objective of this study was to acquire more details of microstructure changes in cork spot disordered pear fruit “Akizuki” (*P. pyrifolia*) using the high-resolution X-ray CT analysis. Results of the study are expected to provide useful information for the control of cork spot disorder for orchard managers and will be valuable for further exploration of the mechanism of the development of cork spot disorder.

## Materials and Methods

Fifteen-year-old “Akizuki” trees grafted on *Pyrus betulaefolia* rootstocks were used for this study at the Experimental Station of Qingdao Agricultural University located at 36°19′N and 120°23′E in the Shandong Province, China. The trees were planted with the spacing of 2 m within rows and 5 m between rows. Trees were managed with full nutrition and water. The fruit was harvested at 140 DAFB for the experiment, which is considered a maturity time point.

### Analysis of Fruit Quality

In total, 10 healthy and 10 cork spotted fruits were used for quality analysis. Fruit attribute parameters such as single fruit weight, vertical length, horizontal length, firmness, soluble solids, and titratable acidity were measured according to the study by Cui et al. (2020). The fruit transverse sections and longitudinal sections were stained with 0.5% safranine solution for 2 min at room temperature and then rinsed with distilled water for 2 min before being photographed by a digital camera (Canon 70D, Japan).

### X-Ray CT Scanning

A nanoVoxel-3502E system (Sanying Precision Instruments Co., Ltd., Tianjin) was utilized to scan both intact fruit and flesh pieces. Samples were rotated on the stage at an increment of 0.4° over a total of 360° at room temperature. The rotating stage was 250 mm away from the X-ray source and 700 mm away from the detector. For intact fruit scanning, the fruit was positioned with the calyx-end to the stem-end axis parallel with the scanner axis of rotation. Transverse and longitudinal section slices were generated from the shadow projections using the Feldkamp reconstruction algorithm (Feldkamp et al., 1984). To obtain high-resolution observations, flesh pieces that were 1 cm away from the cork spot area on the equatorial plane were sampled for scanning. The scanning settings for both types of samples are shown in Table 1.

**Table 1:** Settings of X-ray scanning used in this study.

**Sample type**		**SOD/ODD (mm/mm)**	**Pixel size (μm)**	**Voltage (kV)**	**Current (μA)**	**Scanning time (h)**	**Detector**	**Penetration rate (%)**
Intact fruit	Healthy	169.36/101.44	79.4	80	70	1.5	Large field	50
	Cork spotted	169.36/101.56	79.4	80	70	1.5	Large field	50
Flesh piece	Healthy	21.36/7.76	0.5	50	40	4.5	Optical coupler	50
	Cork spotted	19.35/8.26	0.5	50	40	4.5	Optical coupler	50

*SOD, source origin distance; ODD, origin detector distance*.

### Image Processing

The 16-bit slice images were obtained and then reconstructed into 3D images using the system-supplied reconstruction software with a filtered back-projection algorithm (Feldkamp et al., 1984) after X-ray scanning. To facilitate segmentation, the global thresholds of the grayscale limit were defined to separate pores (i.e., low X-ray attenuation, resulting in lower grayscale values) and flesh tissues (i.e., higher X-ray attenuation, resulting in higher grayscale values). A threshold of 69.7 and 54.8 for cork spotted fruit and healthy fruit, respectively, was used as a discriminative value for the separation of pores and flesh tissue according to the obvious deep valley in the histogram of gray level frequencies (Duan et al., 2020). The 3D stacks of 8-bit grayscale images were then generated after segmentation. Porosity was defined as the pore volume/area divided by the total volume/area of the analyzed sample. The pore equivalent diameter was the diameter of a sphere of equivalent volume as the irregularly shaped object (Jennings and Parslow, 1988).

Avizo 8.1 software (Thermo Fisher Scientific, China) was applied to skeletonize the network for the analysis of the essential pore network with connectivity information between pores. The skeleton analysis procedure includes the extraction of the filar centerline from the scanning data and segmentation of the distance image mapping. Later, the network model was refined, the connection voxel was retained, and the voxel skeleton was converted into a spatial graphics object. Visual inspection of 3D images was conducted using VoxelStudio Max 3.0 software (Volume Graphic, GmbH, Heidelberg, Germany).

### Measurement of Gene Expression

Three time points of the fruit were sampled as follows: 87 DAFB (initiation stage of cork spot), 107 DAFB (early stage of the development of cork spot), and 127 DAFB (full development stage of cork spot). Cork spot disorder initiates much earlier inside the fruit than the symptoms show on the surface. According to our field experience, the fruit flesh turning to light green from white is a signal of cork spot initiation. This usually happens around 87 DAFB, and the spotted tissue continues to develop and turns brownish and lignified later. Therefore, to correctly distinguish and sample the healthy and cork spotted fruit at 87 and 107 DAFB, we need to cut the fruit first and select the fruit with greenish or brownish tissues as cork spotted fruit; otherwise, we think they are healthy. Five healthy fruits and five cork spotted fruits were sampled as biological replications, and each fruit was repeated three times.

The quantitative real-time PCR (qRT-PCR) was applied to measure the expression levels of related genes. Total RNA of the fruit was extracted using the RNAprep Pure Plant Kit (polysaccharides and polyphenolics-rich) (Tiangen, China) according to the instructions of the manufacturer. RNA quality was evaluated by both optical density (OD) value (1.9–2.1) at 260/280 nm (NanoDrop 2000C, Thermo Scientific) and electrophoresis in a 1.5% agarose gel with sharp, clear 28S and 18S rRNA bands stained by ethidium bromide according to the study by Cui et al. (2017). cDNA was synthesized using reagent kits (RR047A, Takara, Japan) according to the instructions of the manufacturer. All cDNA was diluted five times before the qPCR analysis. The qPCR was carried out using reagent kits (RR820A, Takara, Japan) in a 15-μl reaction volume according to the instructions of the manufacturer on a LightCycler 480 instrument (Roche, Switzerland). All genes selected for analysis and their specific primers are displayed in Supplementary Table 1. Two controls of no-reverse transcriptase and no-template were included. The transcript levels of each gene were normalized according to the reference gene, using the 2^−ΔΔCT^ method (Livak and Schmitten, 2001).

### Ca^2+^ Localization

Free Ca^2+^ distribution was analyzed using Fluo-4 AM as a fluorescent Ca^2+^ indicator according to the study by Cui et al. (2020). Fruit at 127 DAFB with the full development of cork spot was sliced at specific areas with a razor blade and then incubated in 4-(2-hydroxyethyl)piperazine-1-ethanesulfonic acid (HEPES) buffer (pH = 7.2) at room temperature for 1 h. The sections were incubated in a mixture (1:1) of 10 μmol/L Fluo-4 AM and 25% Pluronic F-127 for 1 h at 37°C in the dark, followed by rinsing with HEPES buffer for 2 min. A laser scanning confocal microscope (Leica TGS SP5, Germany) equipped with an Optiphot microscope (Nikon, Japan) was used to capture the fluorescence signals at an excitation wavelength of 488 nm stimulated by argon ions. An emission wavelength of 516 nm was captured at a scanning speed of 400 Hz. At least 10 samples of each treatment were used for the observation of free Ca^2+^ distribution.

### Statistical Analysis

The SPSS 17.0 software (IBM-SPSS, Armonk, NY, United States) was used for the statistical analysis of the morphometric measurements. Arcsine transformations were conducted on the data, which are expressed as percentages.

## Results

### Fruit Quality Comparison

At maturity, the single fruit weight of cork spotted fruit (521 g) was significantly greater than that of healthy fruit (471 g) (Table 2). There was no significant difference in fruit vertical length between healthy and cork spotted fruit, while cork spotted fruit had a greater horizontal length (105 mm) than healthy fruit (92 mm). A similar level of fruit firmness was found between healthy and cork spotted fruit. For both soluble solids content and titratable acidity, there were no significant differences between cork spotted and healthy fruit (Table 2).

**Table 2:** Comparison of fruit quality between healthy and cork spotted “Akizuki” fruit.

**Fruit type**	**Fresh weight (g)**	**Horizontal length (mm)**	**Vertical length (mm)**	**Fruit firmness (kg. cm^**−3**^)**	**Soluble solids (Brix^**°**^)**	**Titratable acidity (%)**
Healthy	471 ± 20b	92 ± 4*b*	88 ± 3a	8.1 ± 0.4a	13.7 ± 1.0a	0.83 ± 0.1a
Cork spotted	521 ± 21a	105 ± 4*a*	85 ± 3a	8.2 ± 0.5a	13.2 ± 1.1a	0.78 ± 0.1a

*The data are presented as means ± SE, n = 50. Different letters in the same column indicate significant differences at a level of P < 0.05 by the Student's t-test*.

### Fruit Appearance Observation

**Figure 1:**
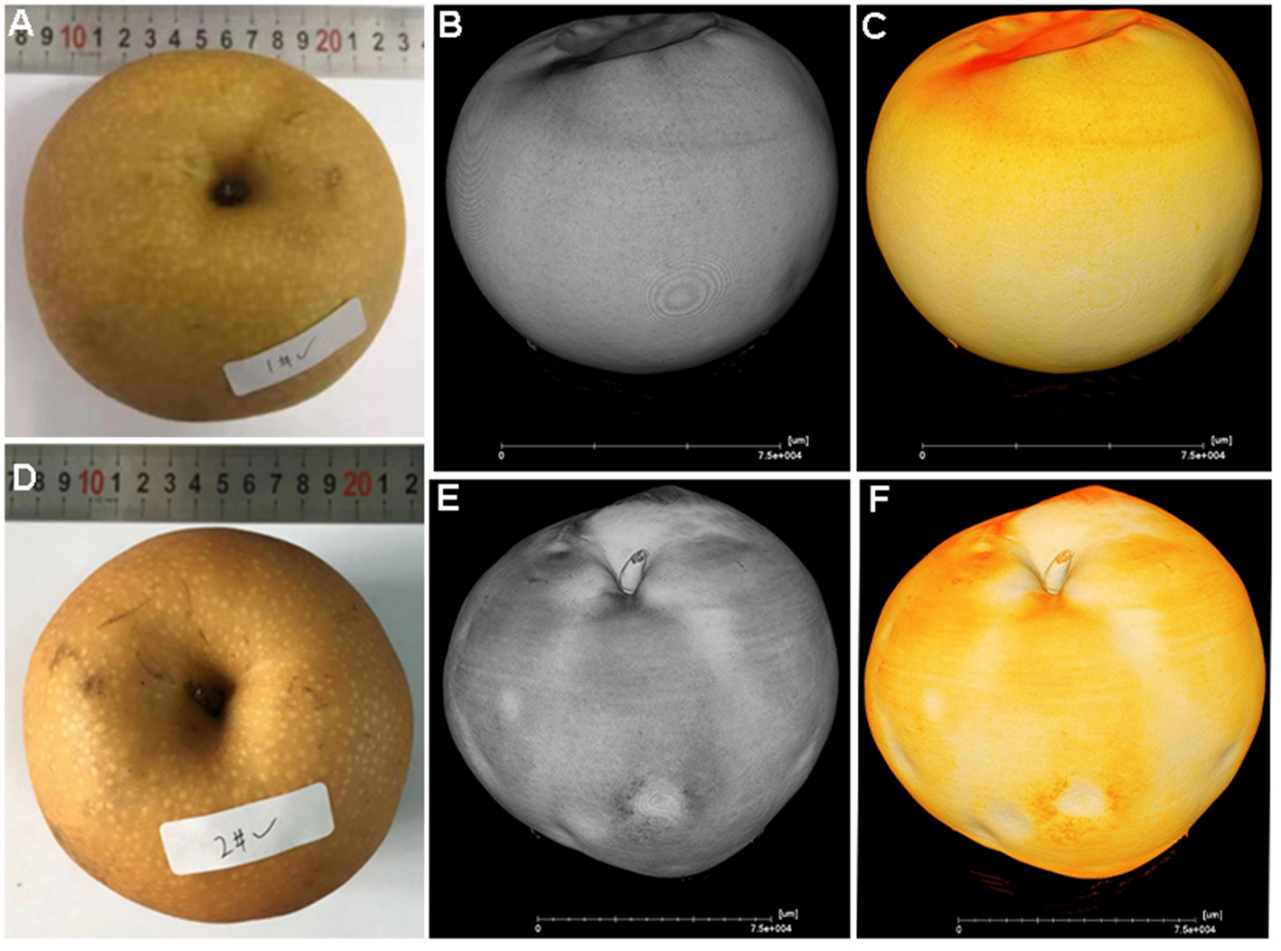
Comparison of the appearance of healthy and cork spotted “Akizuki” fruit at harvesttime. **(A)** Healthy fruit. **(B,C)** Gray and yellow rendering of healthy fruit with X-ray scanning, respectively. **(D)** Cork spotted fruit. **(E,F)** Gray and yellow rendering of cork spotted fruit with X-ray scanning, respectively.

At harvest time, all fruit had a similar appearance, and it was difficult to discriminate between healthy (Figure 1A) and cork spotted fruit (Figure 1D) through visual inspection with the naked eye. From both the gray and yellow 3D images reconstructed from X-ray scanning, the healthy fruit had a smooth surface (Figures 1B,C). However, both the gray and yellow 3D images of the cork spotted fruit showed clearly collapsed pitting on the surface (Figures 1E,F), which was not obvious through normal visual inspection. This indicated that X-ray scanning could discriminate between healthy and cork spotted fruit surfaces at a more accurate level.

### Fruit Cork Spot Distribution Analysis

**Figure 2:**
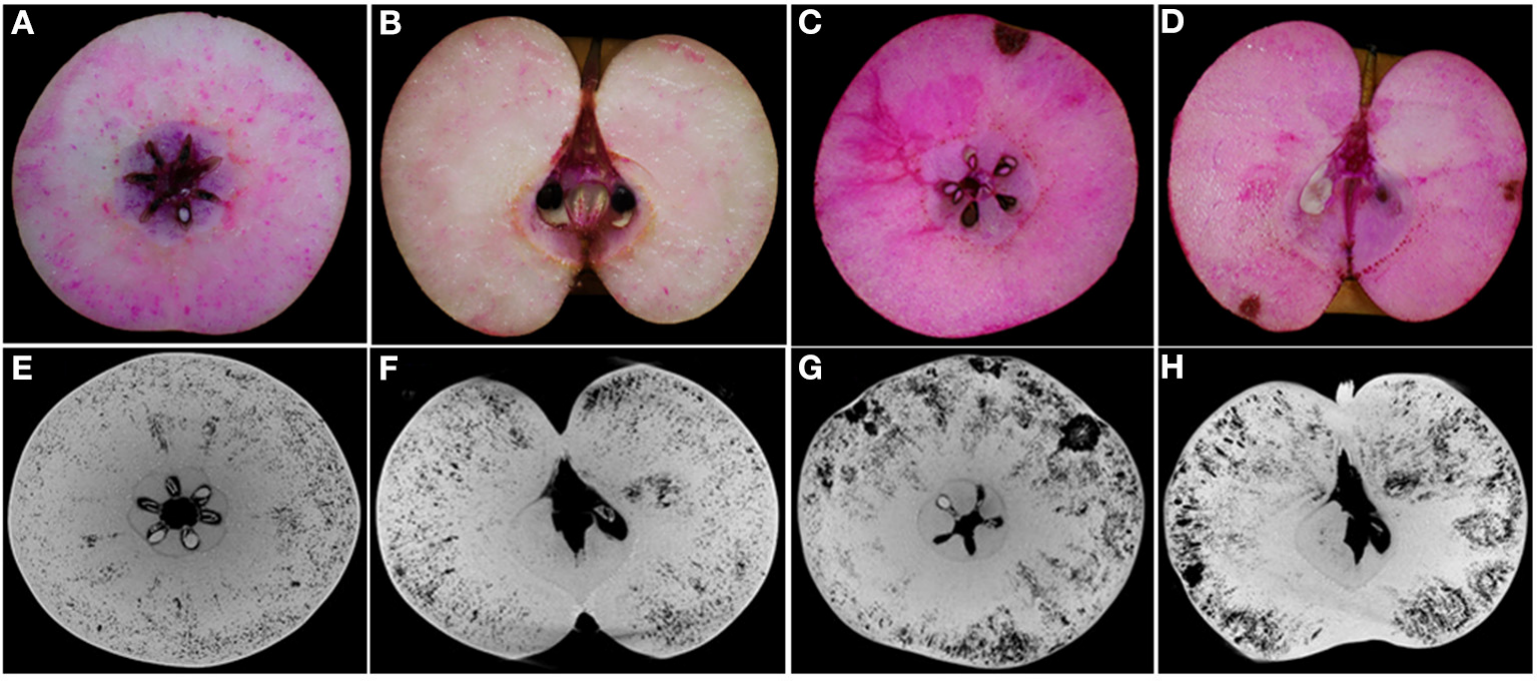
Observation of cork spot distribution in “Akizuki” fruit at harvesttime. **(A,B)** Transverse and longitudinal sections of healthy fruit stained by safranine solution, respectively. **(C,D)** Transverse and longitudinal sections of cork spotted fruit stained by safranine solution, respectively. **(E,F)** Transverse and longitudinal segments of healthy fruit extracted from the X-ray scanning data, respectively. **(G,H)** Transverse and longitudinal segments of cork spotted fruit extracted from the X-ray scanning data, respectively.

From experience in the field over consecutive years, the severity of cork spot disorder varies from year to year depending on the climate and tree growth conditions. In cork spotted fruit, necrotic, and desiccated flesh tissue is distributed close to the fruit skin on the equatorial plane (Figure 2C), while the core and the areas surrounding the core showed a rare distribution of cork spot (Figures 2C,D). The observation on the longitudinal section showed that cork spot distribution is more prone on the calyx-end of the fruit (Figure 2D). Red dots stained by safranine indicate cellulose, which was more present in cork spotted fruit (Figures 2C,D) than in healthy fruit (Figures 2A,B). The segments of both the equatorial plane and the longitudinal section from X-ray scanning were extracted for cork spot distribution analysis (Figures 2E–H). The dark background on the grayscale images was recognized as pores (i.e., lower grayscale values), and the light objects were recognized as tissue (i.e., higher grayscale values). On the equatorial plane, smaller pores (i.e., less density of the dark background) were homogeneously distributed in the healthy fruit (Figure 2E), while larger pores (i.e., high density of the dark background) were observed in the cork spotted fruit (Figure 2G). On the vertical segment, there were fewer and smaller pores in the healthy fruit (Figure 2F), while there were larger and more pores in the cork spotted fruit (Figure 2H). In accordance with the histological observation, the cork spot (i.e., bigger and stronger pores) was distributed close to the fruit skin and the calyx-end of the cork spotted fruit (Figures 2G,H) based on the X-ray scanning analysis. This confirmed the reliability of X-ray scanning for the microstructure analysis of cork spot disordered fruit.

### Porous Structure Analysis of the Whole Fruit

**Figure 3:**
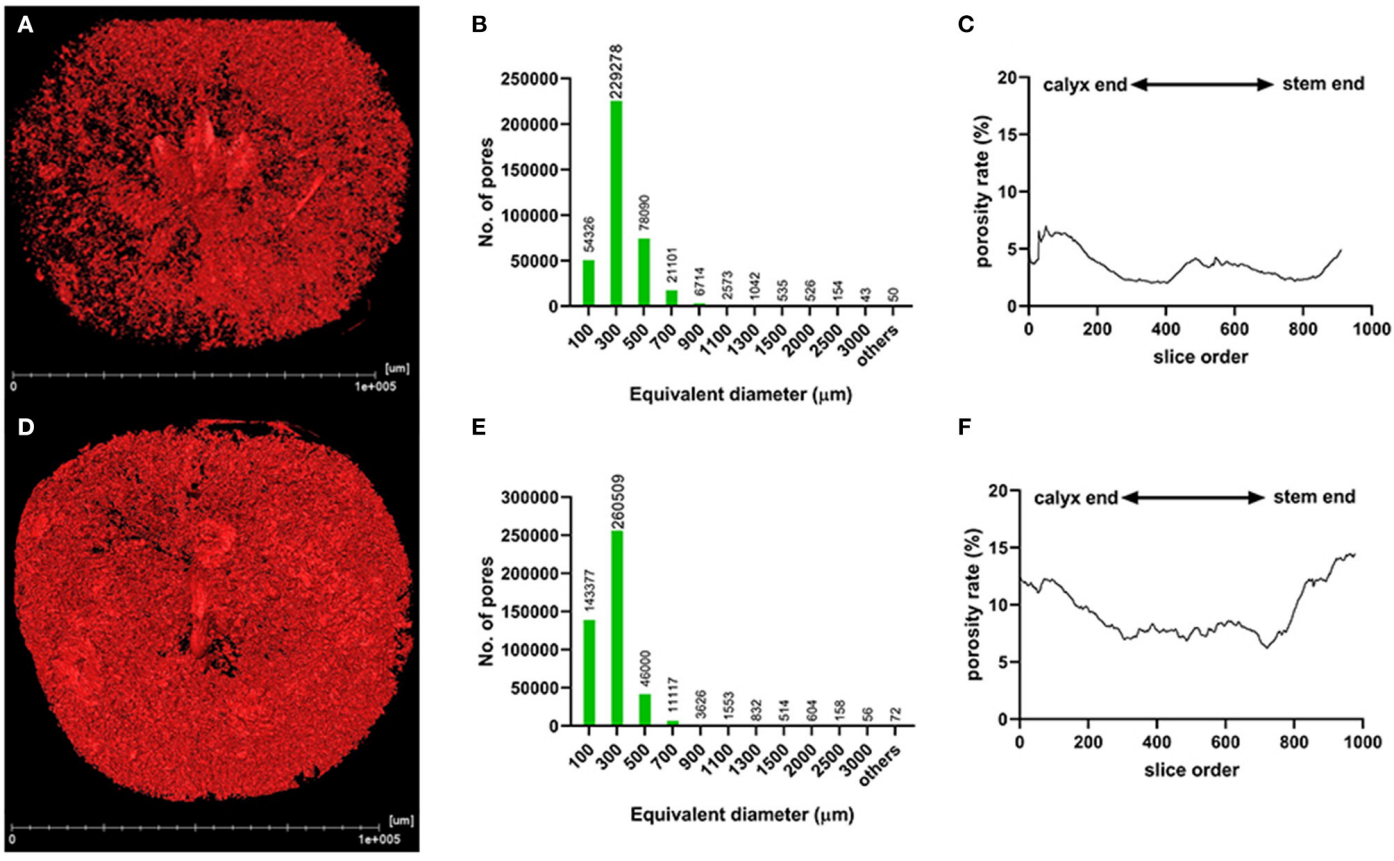
Microstructure analysis of whole “Akizuki” fruit at harvesttime. **(A,D)** Renderings of X-ray scanning of healthy and cork spotted fruit, respectively. Red dots represent pores based on the grayscale value threshold. **(B,E)** Histograms of the pore number and size in healthy and cork spotted fruit, respectively. **(C,F)** Porosity analysis of healthy and cork spotted fruit, respectively. The segmentation of the X-ray scanning data was conducted from the calyx-end to the stem-end of the fruit.

The segmentation of the whole fruit X-ray scanning data was conducted for the porous structure analysis. By stacking the transverse slices from the calyx-end to the stem-end of the fruit, the 3D image of the whole fruit was assembled (Figures 3A,D). The red dots represent the voids based on the threshold values and object density and size (Figures 3A,D). From the whole visual comparison, the cork spotted fruit (Figure 3D) contained several voids inside the fruit than the healthy fruit (Figure 3A). The most frequent pore size was 300 μm in both the healthy and cork spotted fruit (Figures 3B,E) with an amount of 229,278 and 260,509, respectively. Pores measuring 100 μm had an amount of 54,326 and 143,377 in the healthy and the cork spotted fruit, respectively. Other pore sizes (ranging from 500 to 3,000 μm) were more observed in the healthy fruit than in the cork spotted fruit (Figures 3B,E). The average pore diameter was 240 μm in healthy fruit and 260 μm in cork spotted fruit (Table 3). The average number of pores in the healthy fruit was 0.20 mm^−3^, which was significantly less than the cork spotted fruit (0.58 mm^−3^) (Table 3). For the porosity analysis, the porosity of the healthy fruit was high at the calyx-end, decreased toward the middle of the fruit, and then increased close to the stem-end (Figure 3C). In the cork spotted fruit, there were higher levels of porosity (more than 10%) on both the ends of the fruit compared with the middle portion of the fruit (Figure 3F). There was a significant difference in porosity between the healthy fruit (3.52%) and the cork spotted fruit (9.37%) (Table 3).

**Table 3:** Microstructure analysis of healthy and cork spotted “Akizuki” fruit using the segmentation of the X-ray scanning data of the whole fruit.

**Fruit type**	**No. of pore (mm^**−3**^)**	**Porosity (%)**	**Average pore diameter (μm)**
Healthy	0.20 ± 0.05b	3.52 ± 0.51b	240 ± 20a
Cork spotted	0.58 ± 0.08a	9.37 ± 1.21a	260 ± 18a

*The data are presented as means ± SE, n = 5. Different letters in the same column indicate significant differences at a level of P < 0.05 by the Student's t-test*.

### Porosity Analysis of the Cork Spot Area

**Figure 4:**
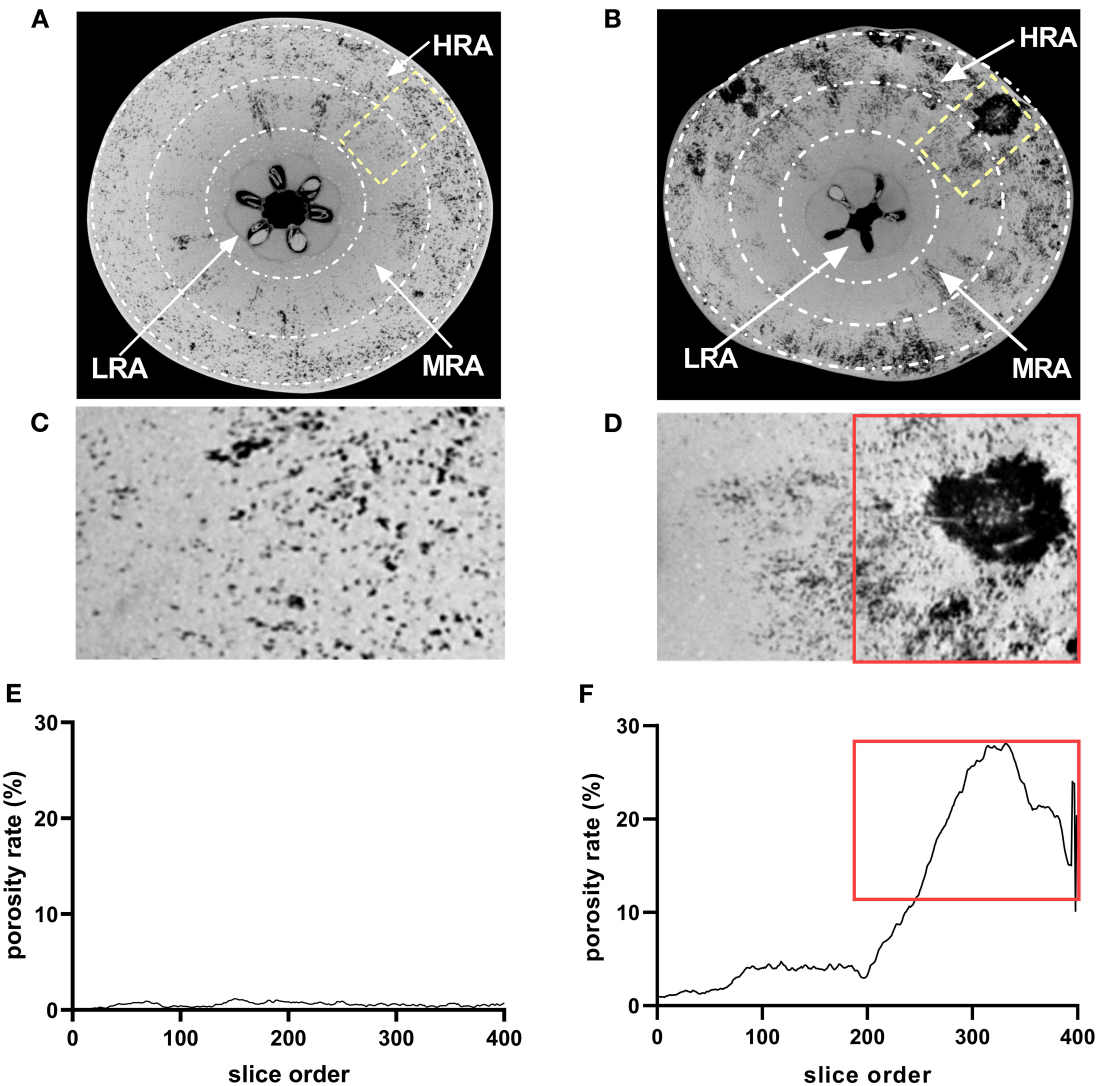
Microstructure analysis of specific areas of “Akizuki” fruit. **(A,B)** Equatorial plane of healthy and cork spotted fruit, respectively. The HRA, MRA, and LRA were defined as the high-risk area with cork spot, the middle-risk area with cork spot, and the low-risk area with cork spot, respectively. **(C,D)** Closer view of **(A)** marked in yellow rectangle and of **(B)** marked in yellow rectangle, respectively. **(E,F)** Porosity analysis of **(C,D)**, respectively. Red rectangles in **(D,F)** represent the tissues and their corresponding porosity.

Based on the histological observation and the porosity analysis of the whole fruit, three different zones of the fruit were identified as follows: the high-risk area (HRA) with cork spot (outer mesocarp), the middle-risk area (MRA) with cork spot (middle portion of the mesocarp), and the low-risk area (LRA) with cork spot (inner mesocarp) (Figures 4A,B). The segmentation of both HRA and MRA scanning data was conducted for the more specific analysis of porosity. For the healthy fruit, the porosity stayed at a low level (<1.5%) (Figures 4C,E), even though it contained the HRA and MRA. While in the HRA and MRA of the cork spotted fruit, a much higher porosity level was found compared with the healthy fruit (Figures 4D,F). For the HRA of the cork spotted fruit, the porosity stayed at a high level of 15–30% (Figures 4D,F, marked with a rectangle). Even though there was no visual cork spot in the MRA of the cork spotted fruit, the porosity level was still close to 5% (Figure 4F), which was much higher than that of the healthy fruit (Figure 4E).

### Three-Dimensional Network Skeleton Analysis of HRA and MRA

**Figure 5:**
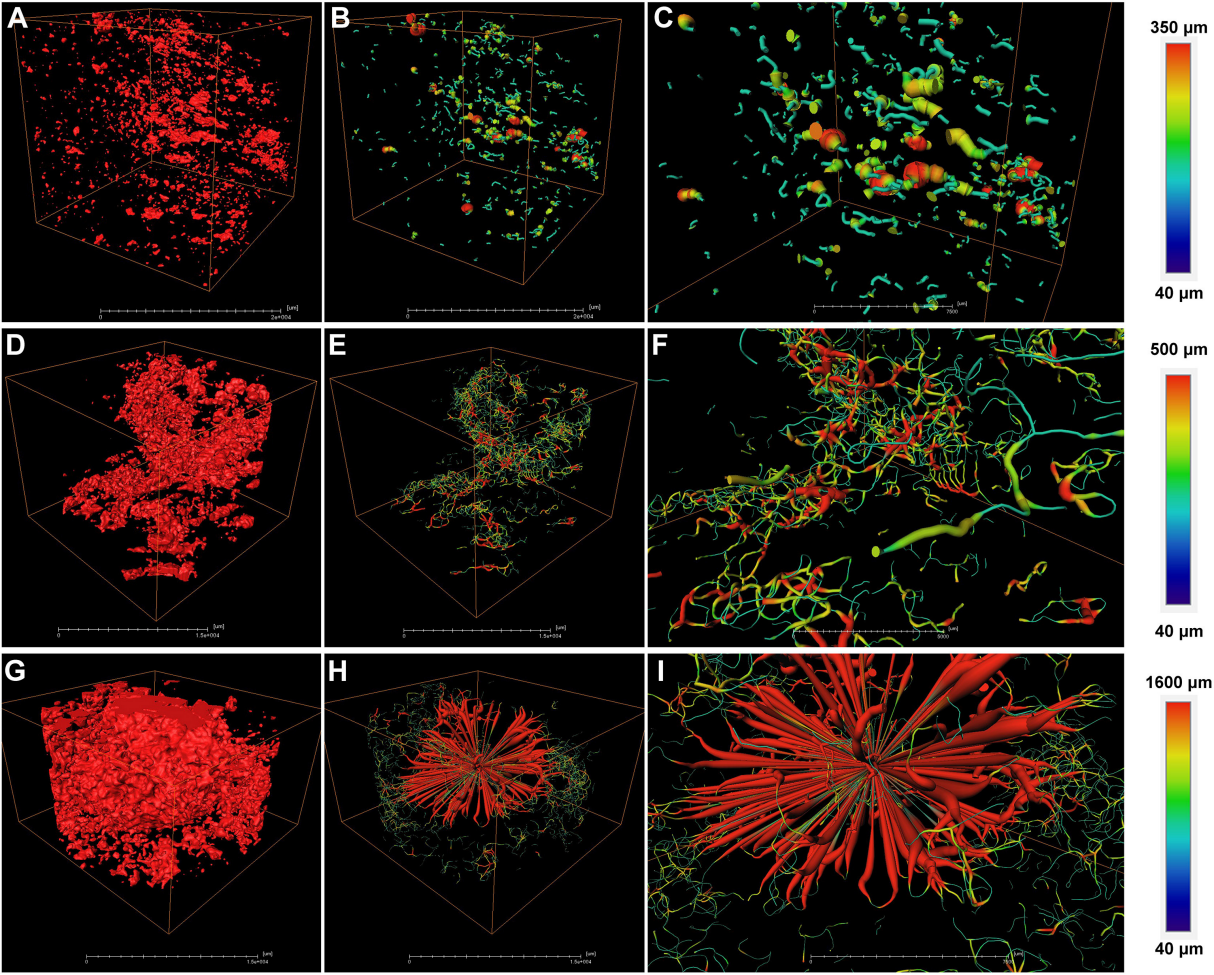
Network skeleton analysis of specific areas of “Akizuki” fruit. **(A)** Pore analysis of the HRA (high-risk area with cork spot) and MRA (middle-risk area with cork spot) of healthy fruit. Red dots represent pores based on the grayscale value threshold. **(B)** Reconstructed 3D network model of **(A)**. **(C)** Closer view of **(B)**. **(D,G)** Pore analysis of the MRA and HRA of cork spotted fruit, respectively. Red dots represent pores based on the grayscale value threshold. **(E,H)** Reconstructed 3D network models of **(D,G)**, respectively. **(F,I)** Closer view of **(E,H)**, respectively. Color bars on the right indicate pore thickness from narrow (blue) to wide (red).

To further analyze the structural properties inside the fruit associating with cork spot, a 3D geometric model was built for a small piece of flesh (2.5 × 1.5 × 1.0 cm) on the equatorial plane containing both the HRA and MRA (Figure 5). Any two voxels of pore volume were connected to build the network model if they shared a face, an edge, or a corner. For the healthy fruit, the HRA and MRA scanning data were used together to build a 3D network (Figures 5A–C). From a general view, the healthy fruit had fewer pores (red dots in Figure 5A) than the cork spotted fruit (red dots in Figures 5D,G), which was consistent with the results in the whole fruit analysis (Figures 3A,D). A low degree of connectivity was found in the pore network of the healthy fruit (Figures 5B,C). The pore channels were based on their thickness. The average length of a pore channel was 119 μm in the healthy fruit (Table 4). The average diameter and average length of a pore throat (an intersection of pores) were 64 and 210 μm, respectively (Table 4). The number of coordinated pores in the healthy fruit was zero, indicating a less branched pore network. Since the porosity of both the MRA and HRA in the cork spotted fruit was relatively high, their network skeleton analysis was separated. In the MRA of the cork spotted fruit, a relatively high density of pores and the highly branched pore channels were observed (Figures 5D–F). The average length of pore channels was 160 μm, remarkably higher than that of the healthy fruit (Table 4). The average length and average diameter of pore throats were 226 and 77 μm, respectively, in the MRA of cork spotted fruit (Table 4). The average and largest number of coordinated pores were 2 and 16, respectively (Table 4). For the HRA of the cork spotted fruit, the average length of a pore channel (161 μm) was similar to that of the MRA in the cork spotted fruit (Table 4). However, the average length and average diameter of a pore in the HRA were remarkably greater (i.e., 268 and 100 μm, respectively) than that of the MRA of the cork spotted fruit (Table 4). This difference was also shown in the 3D network model (Figures 5H,I). A pore network with more red indicated the greater size of the pore channels. The average and largest coordinated number of pores in the HRA of the cork spotted fruit were 2 and 48, respectively (Table 4), indicating the highly branched and connected pore network (Figure 5I).

**Table 4:** Analysis of the morphometric parameters in different areas of healthy and cork spotted fruit under the reconstructed 3D network model.

**Sample type**	**Average length of pore throat (μm)**	**Average diameter of pore throat (μm)**	**Average length of pore (μm)**	**Average no. of coordinated pore**	**Largest no. of coordinated pore**
**Different Areas of Fruit Flesh**					
MRA+HRA of healthy fruit	210 ± 16b	64 ± 5c	119 ± 10b	0	0
MRA of cork spotted fruit	226 ± 17b	77 ± 6b	160 ± 11a	2 ± 0.1a	16 ± 2b
HRA of cork spotted fruit	268 ± 20a	100 ± 8a	161 ± 12a	2 ± 0.1a	48 ± 4a
**Fruit Core**					
Healthy fruit	548 ± 41a	204 ± 18b	698 ± 56a	2 ± 0.1b	5 ± 0.2b
Cork spotted fruit	589 ± 45a	269 ± 21a	497 ± 41b	3 ± 0.1a	12 ± 1a

*MRA, middle-risk area; HRA, high-risk area. The data are presented as means ± SE, n = 5. Different letters in the same column indicate significant differences at a level of P < 0.05. For the significant difference analysis of pore parameters, the Tukey's test was applied for the fruit flesh, and the Student's t-test was applied for the fruit core*.

### Structure and Network Analysis of the Fruit Core

**Figure 6:**
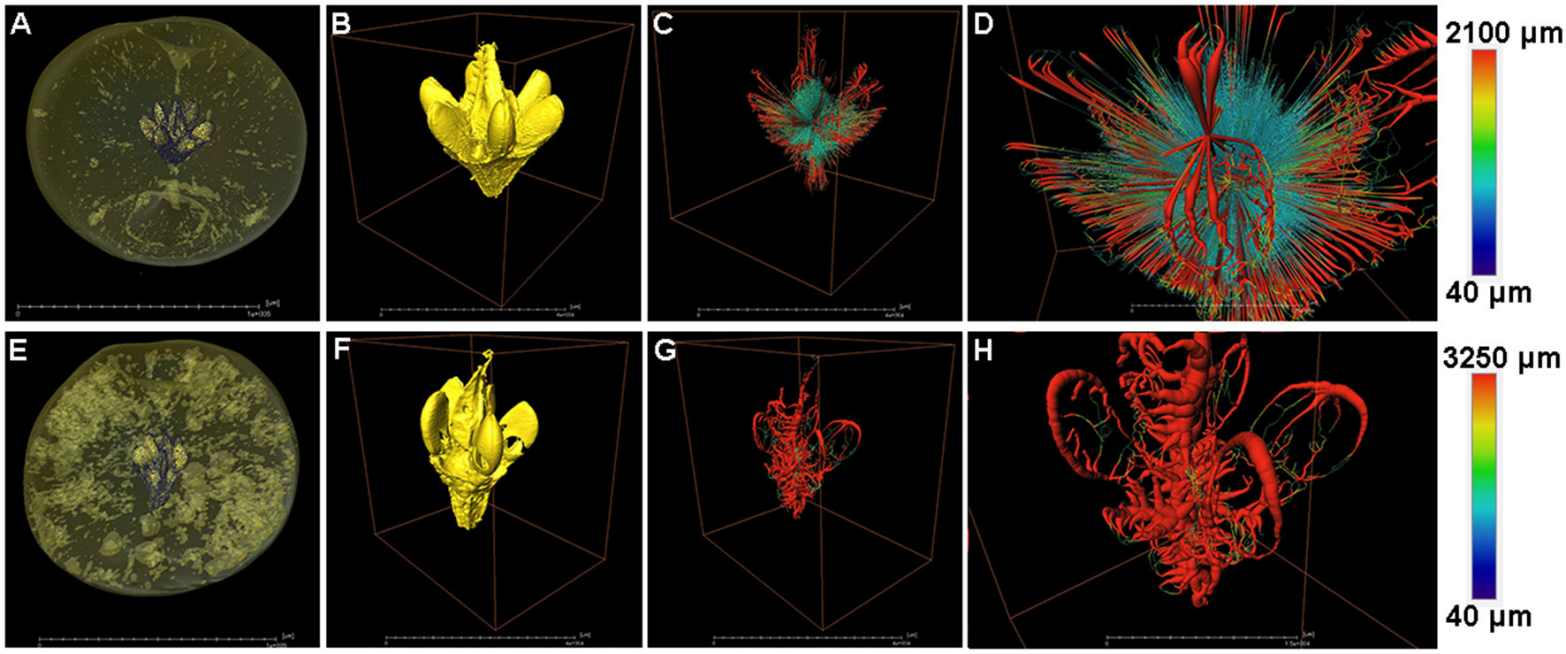
Structure and network skeleton analysis of “Akizuki” fruit core. **(A,E)** Renderings of X-ray scanned healthy and cork spotted fruit, respectively, where the fruit core was distinguished by the grayscale values. **(B,F)** Reconstructed 3D model of the healthy fruit core and cork spotted fruit core, respectively. **(C,G)** Reconstructed 3D network model of **(B,F)**, respectively. **(D,H)** Closer view of **(C,G)**, respectively. Color bars on the right indicate pore thickness from narrow (blue) to wide (red).

The fruit core shape and porous structure were also analyzed in both the healthy and cork spotted fruit (Figure 6). From the whole fruit observations, the healthy fruit core could be clearly separated from the flesh based on the grayscale values (Figure 6A), while the cork spotted fruit core showed a similar texture property to the flesh and could not be distinguished based solely on the grayscale values (Figure 6E). After extracting the scanning data of the core from the fruit and reconstructing it through 3D rendering, the healthy fruit core had an intact shape with a smooth surface (Figure 6B), while the cork spotted fruit core had a damaged shape and rough surface (Figure 6F). For the network connectivity analysis, the healthy fruit core had longer and narrower pore channels (Figures 6C,D) compared with those of the cork spotted fruit core (Figures 6G,H). The average pore channel length was 698 μm in the healthy core and 497 μm in the cork spotted core (Table 4). Both the average length and average diameter of the pore throats were similar between the healthy core (i.e., 548 and 204 μm, respectively) and the cork spotted core (i.e., 589 and 269 μm, respectively) (Table 4). The cork spotted core had a highly branched network structure (Figure 6H) with an average coordinated pore number of 3 and the largest coordinated pore number of 12 (Table 4). In the healthy fruit core, the average coordinated pore number was 2, and the largest coordinated pore number was only 5 (Table 4), which was accordant with its 3D network image with less branches and a lower degree of connectivity (Figure 6D).

### High-Resolution Analysis of the Flesh Microstructure

**Figure 7:**
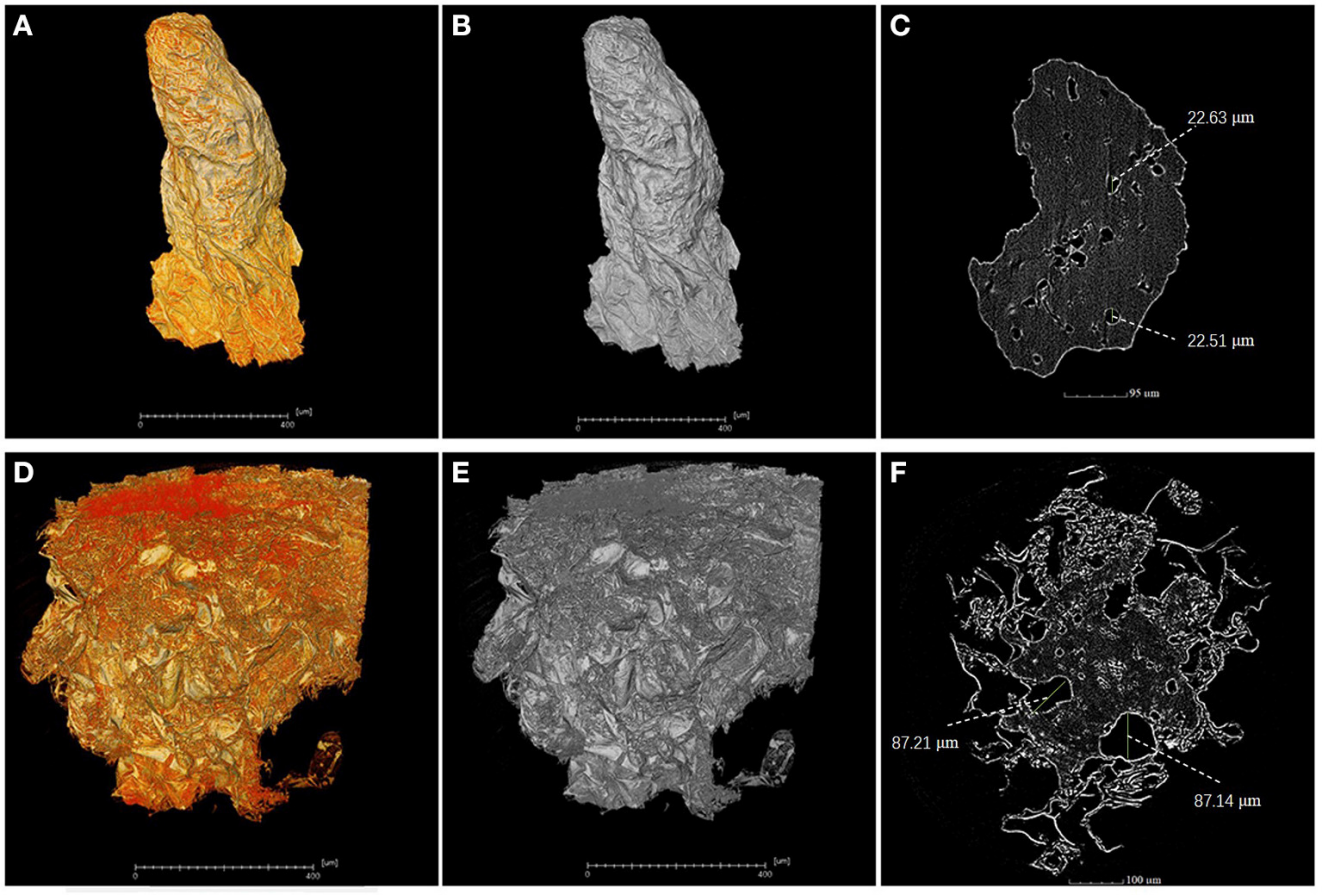
High-resolution analysis of flesh microstructure of “Akizuki” fruit. **(A,B)** Yellow and gray reconstructed 3D images of flesh from the MRA (middle-risk area with cork spot) of the healthy fruit, respectively. **(C)** Segment of the X-ray scanning of the healthy fruit flesh. **(D,E)** Yellow and gray reconstructed 3D images of flesh from the MRA of cork spotted fruit, respectively. **(F)** Segment of the X-ray scanning of the cork spotted fruit flesh.

A piece of flesh from the MRA without cork spot in both healthy and cork spotted fruit was used for the high-resolution observation by X-ray scanning. Under high-resolution observation (pixel size 0.5 μm), the flesh from the healthy fruit had higher grayscale values (Figures 7A,B) than that observed from the cork spotted fruit (Figures 7D,E). In further segmentation analysis, the healthy flesh had a much smaller pore diameter (about 22 μm) (Figure 7C) than that of the cork spotted fruit (about 87 μm) (Figure 7F). This indicated that even in flesh without cork spot, there were obvious texture differences between healthy and cork spotted fruit.

### Analysis of Gene Expression Related to Ca^2+^ Transport

**Figure 8:**
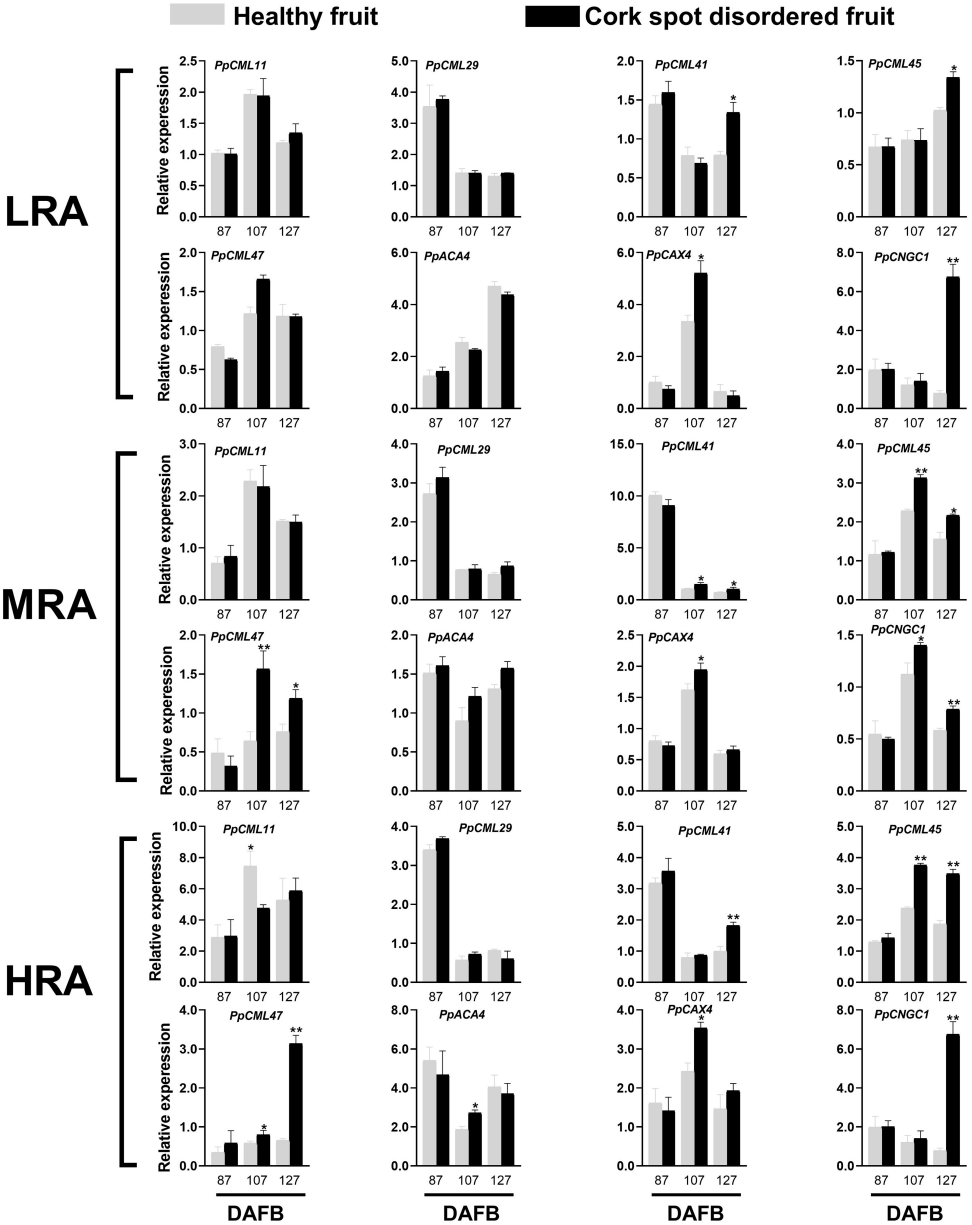
Expression analysis of genes involved in Ca^2+^ transport during fruit development. * and ** indicate the significant differences in the expression within the group at a level of *P* < 0.05 and *P* < 0.01, respectively.

Genes functioning as Ca^2+^ sensors (*PpCML*), Ca^2+^ transporter/channels (*PpCNGC*), Ca^2+^/H^+^ exchangers (*PpCAX*), and Ca^2+^-ATPase (*PpACA*) were selected for expression analysis (Figure 8). Different areas of the fruit were sampled. For the LRA of the fruit, there was no significant difference in the expressions of *PpCML11, PpCML29, PpCML47*, and *PpACA4* from 87 to 127 DAFB between healthy and cork spotted fruit (Figure 8). *PpCML41* had a higher expression level in the cork spotted fruit at 127 DAFB compared with the healthy fruit. *PpCAX4* had a higher expression level in the cork spotted fruit at 107 DAFB than in the healthy fruit but then became similar between healthy and cork spotted fruit at 127 DAFB (Figure 8). For the MRA of the fruit, no significant difference was found in the expression levels of *PpCML11, PpCML29*, and *PpACA4* between the healthy fruit and the cork spotted fruit from 87 to 127 DAFB. *PpCML41* had higher expression levels in the cork spotted fruit than in the healthy fruit at both 107 and 127 DAFB while *PpCML47* had a higher expression level in the cork spotted fruit than in the healthy fruit at both 107 and 127 DAFB. The only significant difference of *PpCAX4* expression was found at 107 DAFB with a higher level in the cork spotted fruit than that in the healthy fruit (Figure 8). For the HRA of the fruit, *PpCML11* had a higher expression level at 107 DAFB in the healthy fruit than that in the cork spotted fruit while no significant difference was found in the expression level of *PpCML29* between healthy and cork spotted fruit from 87 to 127 DAFB. The higher expression level of *PpCML41* was only found at 127 DAFB in the cork spotted fruit compared with the healthy fruit. *PpCML47* had a higher expression level in the cork spotted fruit than in the healthy fruit at 107 DAFB, which then increased drastically and became much higher in the cork spotted fruit at 127 DAFB. A significant difference of *PpACA4* expression was only found at 107 DAFB with a higher level in the cork spotted fruit than in the healthy fruit while *PpCAX4* had a higher expression level in the cork spotted fruit than that in the healthy fruit at 107 DAFB (Figure 8).

### Free Ca^2+^ Distribution

**Figure 9:**
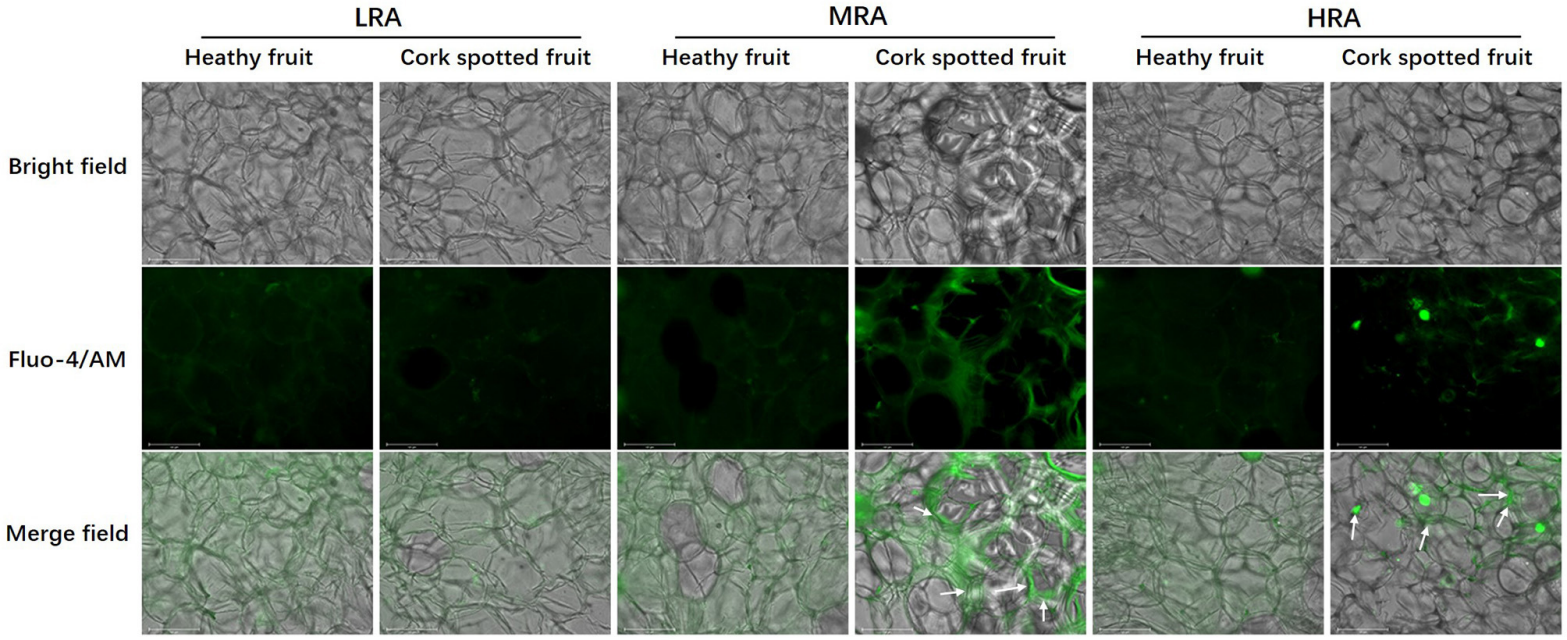
Observation of free Ca^2+^ distribution in different areas of both healthy fruit and cork spotted fruit at 127 days after full bloom (DAFB) by using the laser scanning confocal microscope. Green fluorescence in the merge indicates Ca^2+^ distribution. White arrows indicate the enrichment of the Ca^2+^ signal. Scale bar = 125 μm.

Free Ca^2+^ distribution was analyzed in different areas of both healthy and cork spotted fruit at 127 DAFB (Figure 9). The observation of Ca^2+^ signals was indicated by the green fluorescence in the merged field. For the LRA of the fruit, the Ca^2+^ distribution was homogeneous and diffuse in both healthy fruit and cork spotted fruit with both having a similar intensity of Ca^2+^ signals (Figure 9). For the MRA of the fruit, Ca^2+^ distribution in the healthy fruit had a similar pattern as the LRA while there was an uneven distribution of Ca^2+^ in the cork spotted fruit with several Ca^2+^ signals surrounding the margins of the cells compared with the interior cytoplasm. The Ca^2+^ signals in the cork spotted fruit were stronger than those observed in the healthy fruit (Figure 9). For the HRA of the healthy fruit, the Ca^2+^ distribution pattern was consistent with what was observed in the LRA and MRA in the healthy fruit with a homogeneous and diffuse distribution. However, in the cork spotted fruit, the Ca^2+^ signal intensity was quite uneven. Ca^2+^ signals were centralized to the margins of the cells, similar to what was noted in the MRA, but the Ca^2+^ signal intensity was not as strong as what was observed in the MRA. The cytoplasmic leakage of the HRA may be responsible for the reduced Ca^2+^ signal intensity compared with the MRA, which may be due to the necrosis and high lignification of the HRA (Figure 9).

## Discussion

X-ray CT scanning, which is notably a non-destructive method, has long been developed and validated for the analysis of agricultural food texture during both preharvest and postharvest periods (Wang et al., 2018). However, the application of X-ray CT scanning for the diagnosis and analysis of pear fruit physiological disorders is quite limited (Lammertyn et al., 2003; Muziri et al., 2016; Duan et al., 2020). This study is the first to systematically analyze the cork spot distribution, porous characteristics, network skeleton of different areas, and fruit core integrity and structure in both healthy and cork spotted pear fruit using the high-resolution X-ray CT scanning to explore the causal factors of cork spot disorder in pear.

Our study showed that cork spotted fruit had a greater fruit weight and a larger horizontal length (Table 1), which was consistent with other reports regarding cork spot disorder in pear (Hayama et al., 2017; Duan et al., 2019). Even though cork spotted fruit also had a higher porosity than the healthy fruit (Figure 3), no direct evidence suggested the existence of the correlation between fruit size and porosity. The alteration of the fruit microstructure influences gas exchange/diffusion and water transport inside the fruit and further affects the fruit texture (Kuroki et al., 2004; Mendoza et al., 2007). Both core breakdown in the “Conference” pear (Lammertyn et al., 2003) and mealiness in the “Forelle” pear during the shelf life were found to be associated with higher porosity. Our results showed no correlation between the fruit porosity and fruit texture, including fruit firmness, soluble solids content, and titratable acidity (Table 1). Similar results were also reported in kiwifruit (Cantre et al., 2014a). Ting et al. (2013) correlated firmness with low tissue microporosity in four apple cultivars, and Winisdorffer et al. (2015) found that water content and distribution may influence tissue microstructure, but no correlation was observed between soluble solids content and porosity. This indicated the complex relationship between fruit microstructure and fruit texture, which was controlled by both genetic and environmental factors.

Unlike “Chili,” “Akizuki” has no obvious cork spotting symptoms on the fruit surface (Figure 1). However, X-ray scanning easily distinguished between healthy and cork spotted fruit and was able to discern the cork spot area inside the fruit, which was accordant with the histological observation (Figure 2). This validated the reliability and power of utilizing X-ray in non-destructive testing as reported in other studies (Mebatsion et al., 2009; Winisdorffer et al., 2015; Cakmak, 2019). Cork spotted “Akizuki” fruit had remarkably more pores and higher porosity in the whole fruit (Figure 3), indicating the degradation of flesh tissue. Even though the selected MRA of the cork spotted fruit for the porosity analysis had no cork spot, it still showed much higher porosity compared with the healthy fruit (Figure 4), suggesting that flesh tissue damage occurs prior to the development of cork spot. Cui et al. (2020) observed the necrotic and browning vascular tissues in cork spotted “Chili” fruit, which supported our observation with X-ray scanning. In apple fruit, dysfunctional xylem in the fruit results in a higher (K+ Mg+ N)/Ca ratio and thus induces the development of bitter pit, a similar physiological disorder (Miqueloto et al., 2014). Water core, another physiological disorder, was found to initiate near the vascular bundles according to the study by Kasai and Arakawa (2010). The long-distance transport of Ca^2+^ in fruit is through the apoplastic pathways, which is along the water mass flow in the xylem (Gilliham et al., 2011). Therefore, normal xylem function influences the Ca^2+^ distribution and delivery within the whole fruit. An early loss of xylem function in “Catarina” apple altered the Ca^2+^ content in the fruit and resulted in the higher incidence of bitter pit compared with “Fuji” (Miqueloto et al., 2014), and the Ca^2+^ content in different parts of the wax apple fruit was associated with its xylem functionality during the stages of fruit development (Chen et al., 2019). Both the previous studies (Miqueloto et al., 2014; Chen et al., 2019) emphasized the influence of xylem function in the development of Ca^2+^-deficiency-related fruit disorders. Even though our study did not test the xylem function directly in the healthy and cork spotted fruit, both the porosity (Figure 4) and reconstructed 3D structure analysis (Figure 5) suggested tissue damage in the MRA and HRA of the cork spotted fruit, which possibly altered xylem function. These findings imply that the functions of water and mineral transport channels in the plant likely influence the development of this type of physiological disorder. In the analysis of the fruit porous network, we observed less branched porous channels in healthy fruit (Figure 5), indicating normal porous microstructure for water and nutrient transport and gas exchange, which is critical for proper fruit growth and storage (Kuroki et al., 2004; Winisdorffer et al., 2015). In the cork spotted fruit, the highly branched porous channels (i.e., a large number of coordinated pores) (Figure 5) were possibly due to the extension and interconnection with adjacent small pores, which further aggravated the breakage of the vascular system as observed by the histological section (Cui et al., 2020).

Even though cork spot rarely localized at the inner mesocarp (i.e., tissues surrounding the fruit core) in “Akizuki,” such as what was observed in the study of “Chili” (Duan et al., 2020), the core of cork spotted fruit was deformed with a highly branched pore channel when compared with healthy fruit (Figure 6). This result was accordant with what was observed in cork spotted “Chili” (Duan et al., 2020) despite the major morphological differences between “Akizuki” and “Chili.” To our knowledge, this study was the first to digitize and reconstruct a fruit core 3D model (Figures 6B,F) and to use the X-ray CT scanning data to analyze the structure and network skeleton. The different texture of the fruit core compared with the fruit flesh may prevent the occurrence of cork spot. Another possibility is that the strong vascular connection between the petiole and the core maintained normal Ca^2+^ transport. The deformation of the core may be caused by parts of the flesh affected by cork spot, but this assumption needs further validation. To our knowledge, our high-resolution observation (i.e., 0.5 μm in pixel size) has been the best resolution utilized so far to analyze the fruit microstructure (Figure 7). The MRA of cork spotted fruit had pore sizes four times larger than that of healthy fruit, further confirming our porosity analysis results with the whole fruit. This indicated that flesh tissue microstructure damage occurred before the initiation of the cork spot disorder.

Together with the long-distance transport of Ca^2+^, the cell-to-cell transmembrane transport of Ca^2+^ mediated by membrane transport proteins, such as probable calcium-binding protein (CML), auto-inhibited Ca^2+^-ATPase (ACA), Ca^2+^ exchanger (CAX), and cyclic nucleotide-gated ion channels (CNGC), determines the intercellular and intracellular distribution of Ca^2+^ (Spalding and Harper, 2011; de Freitas and Mitcham, 2012). Recent researchers have proposed that the intracellular imbalance of Ca^2+^ could be the factor causing cork spot disorder, rather than mere Ca^2+^ deficiency (Duan et al., 2019; Cui et al., 2020). The higher expression of *CAX* was shown to lower the cytoplasmic Ca^2+^ concentration in both tomato (de Freitas et al., 2011) and Arabidopsis (Conn et al., 2012) by increasing the Ca^2+^ transport from cytoplasm to vacuole. Once Ca^2+^ is deposited in vacuoles, it is rarely redistributed (Gilliham et al., 2011). Pear superficial scald and pear hard-end, two disorders possibly related to Ca^2+^ deficiency, were shown to associate with the higher expression of *CMLs* and *CNGCs* (Wang et al., 2017; Li et al., 2020). Our study found the higher expression of *PpCML11, PpCML41, PpCML45, PpCML47, PpCAX4*, and *PpCNGC1* in either the MRA or the HRA of the cork spotted fruit during fruit development (Figure 8), indicating that membrane transport proteins play important roles in the development of cork spot disorder. Moreover, the uneven distribution of free Ca^2+^ only appeared in the MRA and HRA of cork spotted fruit (Figure 9). These two areas also showed significantly different gene expressions (Figure 8) and remarkable differences in porous characteristics in the flesh (Figures 4, 5) compared with the healthy fruit. These results suggest that Ca^2+^ cell-to-cell transport and intracellular allocation highly depend on functional membrane proteins (Winisdorffer et al., 2015) and that an undamaged flesh tissue and an intact cell membrane are critical to prevent the development of cork spot.

Considering both previous studies and our present research, it appears that pear fruit cork spot could be initiated by the dysfunction of Ca^2+^ transport, including long-distance transport inside the fruit and intracellular transmembrane transport. The long-distance transport and intracellular transmembrane transport rely on the vascular system and related membrane proteins, respectively. Programmed cell death is an important means in plant immune systems (Coll et al., 2011), and it is believed to be responsible for the larger pore size in apple fruit (Cantre et al., 2014a). Thus, we hypothesized that programmed cell death damaged cell membrane integrity and further caused tissue breakage, which interrupted normal Ca^2+^ balance in the fruit and induced the occurrence of cork spot. Based on this, we propose that future cork spot studies should further explore the mechanism of this disorder and find effective control strategies in the orchard. Since cork spot is a physiological disorder, there are considerable variations of the disorder incidence from year to year, which is affected by, but not limited to, climate, soil structure, water, nutrition, and cultivation measures. Therefore, future studies exploring the mechanism of cork spot should not focus on just one single external factor. Also, there should be an additional investigation regarding the development of the vascular system during fruit growth and the causal factors of vascular system damage, which may influence the Ca^2+^ transport. Furthermore, studies relating to programmed cell death and membrane integrity damage could be a new point of exploration, especially regarding Ca^2+^-transport-related membrane proteins. The multiomics analysis may also facilitate a better understanding of the underlying molecular regulation.

## Conclusion

X-ray scanning is a powerful and reliable tool for the analysis of the microstructure of cork spotted pear fruit, especially under high-resolution observation. Distinct from previous cork spot-related studies, our investigation of the microstructure of pear fruit improved our understanding of the microstructural characteristics of cork spot disorder from a novel perspective. Cork spotted pear fruit had much higher porosity in the whole fruit and more highly branched pore connectivity, which may have interrupted Ca^2+^ transport and allocation. Vascular system breakage and cell membrane damage likely occurred in the fruit prior to the development of cork spot. Additional studies, especially regarding molecular regulation, should be conducted.

## Data Availability Statement

The original contributions presented in the study are included in the article/Supplementary Material, further inquiries can be directed to the corresponding author/s.

## Author Contributions

ZC and NW conducted the experiment, and ZC prepared the manuscript. YD and XX provided impontant technical help in X-ray scanning. RW and SZ gave useful guidance and valuable discussion. CM conceived the idea and provided financial support. All authors contributed to the article and approved the submitted version.

## Conflict of Interest

XX was employed by Sanying Precision Instruments. The remaining authors declare that the research was conducted in the absence of any commercial or financial relationships that could be construed as a potential conflict of interest.

## Publisher's Note

All claims expressed in this article are solely those of the authors and do not necessarily represent those of their affiliated organizations, or those of the publisher, the editors and the reviewers. Any product that may be evaluated in this article, or claim that may be made by its manufacturer, is not guaranteed or endorsed by the publisher.
