# Supplementary material for: High-Resolution Microstructure Analysis of Cork Spot Disordered Pear Fruit “Akizuki” (Pyrus pyrifolia Nakai) Using X-Ray CT
Source: Front Plant Sci. 2021 Aug 16;12:715124. doi: 10.3389/fpls.2021.715124 (PMC8415714; doi:10.3389/fpls.2021.715124)
Supplement: Supplementary file 1 [file Table_1.DOCX]

Table S1 Genes used for expression analysis

| Gene name/description | Gene ID | Primer name | Primer sequence |
| --- | --- | --- | --- |
| *CML11*  /probable calcium-binding protein | LOC103962500 | PpCML11-F | 5'- GGTCCGACATTCAAGGAGAT -3' |
|  |  | PpCML11-R | 5'-AATCTCCCTCCCCCTTAGTT-3' |
| *PpCML29*  /probable calcium-binding protein | LOC103956977 | PpCML29-F | 5'-CTGAGCATCGAGGAGTTCTT-3' |
|  |  | PpCML29-R | 5'-AGCAATAATCCCCTGGCAAT-3' |
| *PpCML41*  / probable calcium-binding protein | LOC103963160 | PpCML41-F | 5'-ACTGCTCGAACCGGAATAAT-3' |
|  |  | PpCML41-R | 5'-ACATGTACTCACCGATGGAC-3' |
| *PpCML45*  / probable calcium-binding protein | LOC103951055 | PpCML45-F | 5'-GCTTTCCTCCAATCCCAAGA-3' |
|  |  | PpCML45-R | 5'-AAACAGCCCTGTAAGCTCAT-3' |
| *PpCML47*  / probable calcium-binding protein | LOC103931383 | PpCML47-F | 5'-AGGCAGCGGAGATTAGTTAC-3' |
|  |  | PpCML47-R | 5'-GTTCTCATCAAACACGCGAA-3' |
| *PpACA4*  / H/ACA ribonucleoprotein complex subunit 4-like | LOC103961078 | PpACA4-F | 5'-CTGTGGCAGAGAAGGAGAC-3' |
|  |  | PpACA4-R | 5'-TCTCTGTGGCATCCTCTAGT-3' |
| *PpCAX4*  / CAX-interacting protein 4-like | LOC103954326 | PpCAX4-F | 5'-GGAGAAGAGGAGGAGGAGAA-3' |
|  |  | PpCAX4-R | 5'-GAATCAGATGGTGTTGCTGC-3' |
| *PpCNGC1*  / cyclic nucleotide-gated ion channel 1-like | LOC108865341 | PpCNGC1-F | 5′-ATGCTTGCCGGTCATGTTAC-3′ |
|  |  | PpCNGC1-R | 5′-ATTGCCAAATCCGTGCCTTT-3′ |
| *PpActin*  Reference gene | Reference gene | PpActin-F | 5'-CCCAGAAGTGCTCTTCCAAC-3' |
|  |  | PpActin-R | 5'-TTGATCTTCATGCTGCTTGG-3' |
